# Effect of Ramadan Fasting on Stress Neurohormones in Women with Polycystic Ovary Syndrome 

**Published:** 2015-06

**Authors:** Farideh Zangeneh, Reza Salman Yazdi, Mohammad Mehdi Naghizadeh, Nasrin Abedinia

**Affiliations:** 1 Vali-e-Asr, Reproductive Health Research Center, Tehran University of Medical Sciences, Tehran, Iran; 2 Research Institute for Islamic and Complementary Medicine (RICM), Tehran, Iran; 3 Department of Andrology, Reproductive Biomedicine Research Center, Royan Institute for Reproductive Biomedicine, ACECR, Tehran, Iran; 4 Department of Community Medicine, Medical Faculty, Fasa University of Medical Sciences, Fasa, Iran; 5 Maternal, Fetal‐ Neonatal Research Center, Tehran University of Medical Sciences, Tehran, Iran

**Keywords:** Ramadan Fasting, Polycystic Ovary Syndrome (PCOS), Cortisol, Adrenaline, Noradrenaline

## Abstract

**Objective:** To determine the effects of Ramadan fasting on serum levels of stress neurohormones in Iranian women with polycystic ovary syndrome (PCOS).

**Materials and methods:** This study was a clinical trial and was performed during July 2011 (month of Ramadan) in Royan institute, Tehran. A total of 40 women who were aged 20-40 years and known cases of PCOS and had no other medical diseases were included in the study. They were divided into two groups as follows: (i) study group (n = 20) who participated in Ramadan fasting and (ii) control group (n = 20) who did not participate in fasting. For evaluating Ramadan’s effect on the level of neurohormones serum level of the following variables were evaluated before and after Ramadan: cortisol, adrenaline (A), noradrenalin (NA), beta-endorphin (β-End), insulin, as well as sex hormones including follicle-stimulating hormone (FSH), luteinizing hormone (LH), and testosterone.

**Results:** In the study group after Ramadan serum cortisol and nor-adrenaline levels were significantly lower than the initial levels obtained at beginning of Ramadan (p < 0.05) as compared to control group.

**Conclusion:** This study indicates that Ramadan fasting decreases stress neurohormones in women with PCOS.

## Introduction

As for the history of fasting in Islam; for the first time, refers to the year of six A.H. The Holy Prophet (SAW) after peace of Hadibiah departed for Medina and performed the Ramadan ceremonies and then, Shawwal in there. Islam considers especial respect for fasting practice. In the holy Quran, God says that:” believers, fasting are decreed upon you as it was decreed on those before you; perchance you will be cautious”. According to this verse, fasting had existed even in the former religions (1). Studies in the recent decade have indicated that proper life style is the need for healthy human society. Islamic fasting with its spiritual property is the most significant and responsible model to maintain mental and physical health. In Ramadan, food and fluid ingestion is restricted from the pre-sunrise to post-sunset hours for one complete month each year. It is obligatory for the healthy adult Muslims to abstain from eating, drinking, and smoking each day from dawn to sunset during the month of Ramadan (2). Studies in recent decades have shown that lifestyle intervention improves body composition and that Ramadan fasting can be an excellent practice for Muslims who are trying to improve their moral character and habits by what they believe to be virtue. Changes in the nyctemeral pattern observed during Ramadan include diurnal fasting from sunrise to sunset with delayed and shortened periods of sleep (3). 

Polycystic ovary syndrome as a complex, multifaceted, heterogeneous disorder affects 4% to 18% of reproductive-aged women and is associated with reproductive, metabolic and psychological dysfunctions (4). The clinical features include reproductive manifestations such as reduced frequency of ovulation and irregular menstrual cycles, reduced fertility, polycystic ovaries on ultrasound, and high male hormones such as testosterone which can cause excess facial or body hair growth and acne. Previous studies have shown that PCOS may cause some psychological disorders. The relationships between the psychological health aspects and the clinical characteristics of PCOS are not yet clear. PCOS affects quality of life through making anxiety and depression symptoms worse either due to the features of PCOS or the diagnosis of a chronic disease (5). In studies by Adaliand Hirschberg (6), they have suggested that treatment of PCOS should tackle both physical and psychological discontents. This is because psychological distress reduces motivation, and yet good motivation is the key to agreement with medication and dietary management of PCOS (7).

In this study, we have intended to determine the effects of Ramadan fasting on stress neurohormones cortisol, cathecolamines and sex hormones in women with polycystic ovary syndrome.

## Materials and methods

In this clinical trial study, all PCOS women were visited in Royan Institute, Tehran, Iran. All the procedures were also performed at the Obstetrics and Gynecology Department of Royan Institute during July 2011 (month of Ramedan). The diagnosis of PCOS was made according to the joint criteria of the European Society of Human Reproduction and Embryology (ESHRE) and the American Society of Reproductive Medicine (ASRM) (8). A total of 40 PCOS Iranian women aged 20-40 years without other chronic disease were then recruited. The patients were divided into two groups as follow: (i) study group (n = 20) who participated in Ramadan fasting and (ii) control group (n = 20) who did not participate. At end of Ramadan, 5 ml of a fresh blood sample was taken from each woman who passed one cycle of menstrual period during this month. 

***Clinical and laboratory investigation***

Clinical data including information about hirsutism, acne, menstrual cycle, body mass index (BMI) and pattern of fasting, such as nutrition and sleep, were gathered through a physical examination performed by an assigned physician. The BMI was calculated as weight (kg)/[height (m)]^2^. Furthermore, serum concentrations of adrenaline (A), noradrenaline (NA; Oxidized LDN Elisa, Germany), beta-endorphin (β-End), estradiol (β-EP ELISA Kit, China), cortisol and insulin (ELISA kit, Diametra, Italy) as well as sex hormones including follicle-stimulating hormone (FSH), luteinizing hormone (LH) and testosterone (ELISA kit, Monobind from USA) were measured. A demographic-social questionnaire including age, education, occupation, and duration of illness was also completed through a face-to-face interview. 

***Statistical analysis***

Data presents as mean ± standard deviation (SD) and median. Comparison of symptoms between study groups was done using Chi square test. The concentrations serum of FSH, LH, testosterone, insulin and cortisol were compared between two groups using student’s t-test. For compering the concentrations serum of other hormones, Mann-Whitney test was used. Spearman rank correlation coefficient was used to assess relationship of hormones with each other. All data were analyzed using Statistical Package for the Social Sciences (SPSS; SPSS Inc., Chicago, IL, USA) version 18. A p-value less than 0.05 were considered as a significant level.

***Ethical considerations***

This study was approved by the Ethics Committee of Tehran University of Medical Sciences. 

The study objectives were explained to the patients before they entered the study, and an informed consent was obtained from all.

## Results

This study included 40 PCOS women, of whom 20 individuals belonged to fasting (study group) and 20 individuals belonged ton on-fasting (control group) groups. Mean age values of fasting and non-fasting groups were 28.8 ± 3.67 and 29.4 ± 4.60 years, respectively (p = 0.658). The women belonging to fasting and non-fasting groups weighed 63.9 ± 5.78 kg and 66.3 ± 6.42kg (p = 0.221), respectively. Acne was showed in 6 (30%) women of fasting group and also in 5 (25%) individual of non-fasting group. The findings showed that acne (p = 0.723), hirsutism (p = 0.451), and irregular menstrual period (p = 0.231) were the same for two groups. Socio-demographic information and symptoms of participant’s were presented in table 1.

Woman in study group fasted for more than 25 days, while their mean of fasting days was 26 ± 4 days. Women in fasting group were waking up between 3:15 to 3:45. Before Azan, they ate food and then they were sleeping in average 4.5 ± 2.1 hours. They also had 1.4 ± 1.4 hours sleep at afternoon. Pattern of eating food in fasting group was presented in table 2. 

Biochemical test results showed that there are no significant differences in the serum concentrations of FSH (p = 0.542), LH (p = 0.827) and testosterone (p = 0.683) between two groups after Ramadan (Table 3). Cortisol hormone concentration decreased in fasting group (p = 0.049). It was 11.2 ± 9.3 in non-fasting group and it was 8.2 ± 6.7 in fasting group.

Nor-adrenaline level decreased in fasting group (p = 0.047), while its serum concentration was 1430 ± 404 in non-fasting and 1176 ± 439 in fasting group. Similarly adrenaline level decreased in fasting group, whereas its concentration was not significantly different between two groups (p = 0.151). There are no significant differences regarding β-End (p = 0.543) and insulin (p = 0.818) concentrations between two groups after Ramadan month (Table 4). 

**Table 1:** Socio-demographic information and symptoms of PCOS women

	Ramadan fasting		p value
	No (n = 20)	Yes (n = 20)	
	Mean ± SD	Mean ± S D	
**Age (year)**	**28.80± 3.86**	**29.40± 4.60**	**0.658**
**Age at marriage time (year)**	**21.00± 3.09**	**21.15± 3.91**	**0.894**
**Infertility duration (year)**	**7.55± 4.05**	**6.60± 3.73**	**0.445**
**Weight (kg)**	**66.30± 6.42**	**63.90± 5.78**	**0.221**
**Acne, n (%)**	**5 (25.0%)**	**6 (30.0%)**	**0.723**
**Hirsutism**	**17 (85.0%)**	**14 (70.0%)**	**0.451**
**Irregular menstrual cycle**	**20 (100.0%)**	**17 (85.0%)**	**0.231**

**Table 2:** Eating pattern of PCO women in fasting group at before sunrise, after sunset and dinner

Time of eating	Volume of eating		
	Not eating	Light meal	Full meal
	n (%)	n (%)	n (%)
**Before sunrise **	**3 (15.0%)**	**5 (25.0%)**	**12 (60.0%)**
**After sunset **	**0 (0.0%)**	**8 (40.0%)**	**12 (60.0%)**
**Between sunset and sunrise**	**11 (55.0%)**	**4 (20.0%)**	**5 (25.0%)**

**Table 3:** Biochemical tests results of PCOS women after Ramadan

	Control (n = 20)		Ramadan Fasting (n = 20)		p value
	Mean ± SD	Median	Mean ± SD	Median	
**FSH (mIU/ml)**	**5.60± 1.96**	**5.55**	**5.24± 1.72**	**5.40**	**0.542**
**LH (mIU/ml)**	**8.08± 6.77**	**5.45**	**8.51± 5.46**	**7.10**	**0.827**
**Testosterone (ng/ml)**	**1.73± 1.18**	**1.45**	**1.94± 1.97**	**1.20**	**0.683**
**Cortisol (µg/dL)**	**11.2± 4.7**	**9.3**	**8.2± 4.4**	**6.7**	**0.049**

**Table 4:** Hormonal tests results of PCOS women after Ramadan

Hormones	Control (n = 20)		Ramadan Fasting (n = 20)		p value
	Mean ± SD	Median	Mean ± SD	Median	
**Adrenaline (pg/ml)**	**135.49 ± 97.90**	**110.00**	**98.83 ± 82.96**	**84.00**	**0.151**
**Noradrenaline (pg/ml)**	**1430.30 ± 404.46**	**1503.50**	**1176.15 ± 439.16**	**1273.50**	**0.047**
**Beta endorphin (ng/Lit)**	**233.2 ± 494.6**	**47.7**	**360.2 ± 759.2**	**55.6**	**0.543**
**Insulin (mIU/Lit)**	**19.2 ± 18.2**	**11.5**	**19.7 ± 20.8**	**11.5**	**0.818**

**Figure 1:**
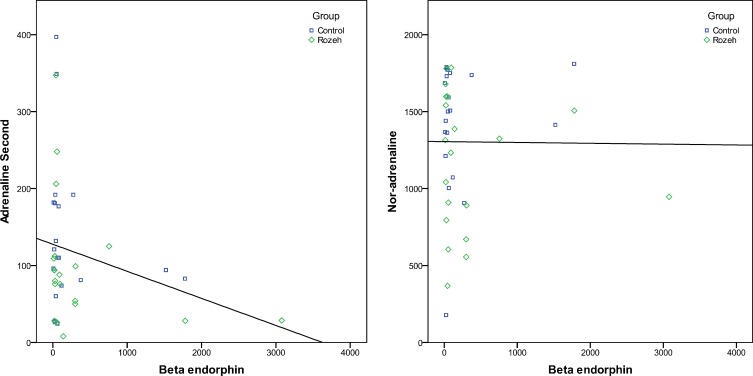
Relationship between beta endorphin level with adrenalin and noradrenalin levels in PCOS women after Ramadan

**Table 5:** Spearman's correlation coefficient between hormones levels in PCOS women after Ramadan

		Cortisol	Adrenaline	Nor-adrenaline	Beta endorphin	Insulin
**Cortisol **	**r**		**0.043**	**0.170**	**-0.030**	**-0.140**
	**p**		**0.793**	**0.294**	**0.856**	**0.390**
**Adrenaline **	**r**	**0.043**		**0.197**	**-0.184**	**-0.057**
	**p**	**0.793**		**0.224**	**0.256**	**0.726**
**Noradrenaline**	**r**	**0.170**	**0.197**		**-0.119**	**-0.328** ^*^
	**p**	**0.294**	**0.224**		**0.464**	**0.039**
**Beta-endorphin**	**r**	**-0.030**	**-0.184**	**-0.119**		**0.142**
	**p**	**0.856**	**0.256**	**0.464**		**0.384**
**Insulin**	**r**	**-0.140**	**-0.057**	**-0.328** ^*^	**0.142**	
	**p**	**0.390**	**0.726**	**0.039**	**0.384**	

There is an inverse correlation between concentrations of insulin and noradrenalin (r = -0.328, p = 0.039). β-End level shows a non-significant inverse correlation with adrenalin (p = 0.256) and nor-adrenalin (p = 0.464) levels (Figure 1). Also adrenalin and noradrenalin levels show to have a non-significant direct correlation (p = 0.224) (Table 5).

## Discussion

In this study, we investigated the circadian variations of serum neurohormones levels which are considered as one of the “biological clock” markers. “The phase and amplitude of these rhythms in physiology and behavior are generated by circadian oscillators and entrained to the 24 hour per day by exposure to the light-dark cycle and feedback from the sleep-wake cycle. The extent to which the phase and amplitude of multiple rhythms are similarly affected during altered timing of light exposure and the sleep-wake cycle has not been fully characterized “(9). Our results show that the concentration values of sex hormones, FSH, LH, testosterone and insulin were unchanged during Ramadan fasting in PCOS women, but stress level related to neurohormones and β-End concentrations decreased. Decreasing in adrenaline and β-End concentrations were not significant. McCartney’s research in 2010 has showed that “the sleep and the endocrine system saved a bidirectional relationship in which depends on each other to regulate different physiological processes including sleep. One of the clearest relationships are that estrogen and progesterone have, that causing changes in sleep patterns associated with the hormonal cycles of women throughout life, from puberty to menopause and specific periods such as pregnancy and the menstrual cycle, including being responsible for some sleep disorders such as hypersomnia and insomnia” (10, 11). By contrast to patterns observed in normal cycles, episodic slowing of GnRH pulse frequency does not regularly occur in PCOS. The studies conducted during the Islamic month of fasting, Ramadan, have showed that many people alter their sleeping habits and stay awake most of the night. Ramadan is a period of sleep deprivation, but many studies have shown an increased interest in recent years in sleep changes and daytime sleepiness during Ramadan. “Moreover, many of those who fast during Ramadan associate this fasting with increased daytime sleepiness and decreased performance. This raises the question of ‘whether Ramadan fasting affects sleep” (12). Therefore, fasting is considered as a religious practice and compared to all other practices is an exceptional one. Whereas, God ordered to Prophet Mohammad; everything that man does is only for himself except fasting, which is done for me, and I shall reward it without any intermediate point (13). This order of the Prophet is considered as confirmation of month of Ramadan, which is known as month of the God’s banquet, while the Prophet Mohammad had mentioned, “This is the God’s holly reception and all are invited” (14). The aim of fasting in month of Ramadan is to establish a proper religious model and life style. Cortisol, a hormone released in stressful situations, when an individual must react to an extraordinary demand that threatens his or her survival, but also known as the hormone of awakening because the peak of release occurs in the morning, although this may be altered in some sleep disorders like insomnia and mood disorders. Roky et al. (2001) reported that “Ramadan fasting on nocturnal sleep, with an increase in sleep latency and a decrease in SWS and REM sleep, were attributed to the inversion of drinking and meal schedule, rather than to an altered energy intake which was preserved in this study” (15). In a study by Bogdan et al. (2005), they showed “that serum concentrations of neuroendocrine hormones like melatonin, steroid hormones (cortisol, testosterone), pituitary hormones (prolactin, LH, FSH, GH, TSH) were documented around the clock at six 4-hourly intervals before Ramadan began and on the twenty-third day of Ramadan (daytime fasting).Statistically significant differences were found in some variables: the nocturnal peak of melatonin was diminished and may have been delayed; there was a shift in the onset of cortisol and testosterone secretion; the evening peak of prolactin was enhanced, FSH and GH rhythmic patterns were affected little or not at all by Ramadan fasting. These data show that daytime fasting; modifications in sleep schedule and psychological and social habits during Ramadan induce changes in the rhythmic pattern of a number of hormonal variables” (16). Our study on PCOS patients during Ramadan fasting showed that, LH serum level had a non-significant increase, while FSH and testosterone levels did not change. 

The study on PCOS patients in South Asians shows adverse affects on their psychological wellbeing and health-related quality of life. Their psychological distress is related to hirsutism rather than to obesity (17). Indian studies on psychological stress using Goldberg's GHQ 28 (General Health Questionnaire) assessed psychological status in ninety nine women with PCOS. The findings of this psychological study showed that 72% had obesity, 70% had hirsutism and 72% had a waist circumference > 88cm. All these variables were statistically significant and Indian women presenting with PCOS had increased psychological distress (18). The clinical signs of PCOS women in Iran showed to be most closely associated with psychological distress which has important implications for the diagnosis and treatment of disorders (19). Some other studies have shown that physical and psychological stressors activate the hypothalamo-pituitary-adrenal axis and lead to marked and persistent increase in serum concentrations of glucocorticoids (20). Molecular mechanisms underlying pathophysiology of PCOS, especially those related to cortisol signaling are poorly understood. Milutinović et al. hypothesized that modulation of glucocorticoid receptor (GR) expression and function, may underlie possible PCOS-related impairment of feedback inhibition of hypothalamic-pituitary-adrenocortical (HPA) axis activity and thus contribute to increased adrenal androgen production in women with PCOS (21). Cortisol production in PCOS women increases in comparison with related levels in healthy women (controls) (22). Hadramyet al. reported that 4 of 10 subjects showed alterations in the cortisol rhythm during the last 2 weeks of Ramadan (23). Our results show adrenaline serum level had an insignificant decrease, but cortisol and noradrenaline serum levels significantly decreased in women with PCOS during Ramadan. It is well known that one of the major neurotransmitters that control LH secretion is noradrenaline. As women with PCOS have significantly higher sympathetic nerve activity than their matched controls, the increased sympathetic outflow may be related to hormonal and metabolic features that may be relevant to the pathophysiology of the syndrome. The central hypothalamic β-End system has a regulatory role in a variety of functions, including reproduction and autonomic function.β-End is a key mediator of changes in autonomic functions (24). Zangeneh et al. (2011) indicated that both sympathetic and opioid systems and interaction between them are effective in processing modeling of PCOS in rat. Interaction of two drugs (clonidine and naltrexone) decreased estradiol, so qualitative analysis showed that corpus lutea and dominant follicles were increased in PCOS rats in comparison with control group (25). Eyvazzadeh et al.’s study postulated this hypothesis that naltrexone administration in conjunction with other established ovulation induction regimens may have beneficial central and peripheral effects. However, there is limited support for naltrexone as a single ovulation induction agent in patients with PCOS (26). In this study, β-End levels showed a non-significant increase in the study group as compared to the related value in the control group. This study showed that Ramadan fasting is a pattern well known to reduce stress hormones in PCOS women. Future studies, specifically in PCOS women, with a larger sample size, and with evaluation of parameters, such as sleep and nutrition, are recommended, just before and after Ramadan. 

## Conclusion

Fasting month of Ramadan is a particular pattern of human lifestyle. It can reduce stress neurohormone and insure the health of the human body and mind. 

## References

[1] Verse 185, Al-Baghara. Quran-e-Karim translated by Qarib.

[2] Reilly T, Waterhouse J (2007). Altered sleep-wake cycles and food intake: the Ramadan model. Physiol Behav.

[3] Ben Salem L1, B'chir S, Bchir F, Bouguerra R, Ben Slama C (2002). Circadian rhythm of cortisol and its responsiveness to ACTH during Ramadan. Ann Endocrinol (Paris).

[4] Moran LJ, Hutchison SK, Norman RJ, Teede HJ (2011). Lifestyle changes in women with polycystic ovary syndrome. Cochrane Database Syst Rev.

[5] Teede H, Deeks A, Moran L (2010). Polycystic ovary syndrome: a complex conditions with psychological, reproductive and metabolic manifestations those impacts on health across the lifespan. BMC Med.

[6] Adali E, Yildizhan R, Kurdoglu M, Kolusari A, Edirne T, Sahin HG (2008). The relationship between clinico-biochemical characteristics and psychiatric distress in young women with polycystic ovary syndrome. J Int Med Res.

[7] Barnard L, Ferriday D, Guenther N, Strauss B, Balen AH, Dye L (2007). Quality of life and psychological well-being in polycystic ovary syndrome. Hum. Reprod.

[8] Rotterdam ESHRE/ASRM-sponsored PCOS Consensus Workshop Group (2004). Revised 2003 consensus on diagnostic criteria and long-term health risks related to polycystic ovary syndrome (PCOS). Hum Reprod.

[9] Dijk DJ, Duffy JF, Silva EJ, Shanahan TL, Boivin DB, Czeisler CA (2012). Amplitude reduction and phase shifts of melatonin, cortisol and other circadian rhythms after a gradual advance of sleep and light exposure in humans. PLoS One.

[10] McCartney RC (2010). Maturation of sleep-wake GnRH secretion across puberty in girls: potential mechanisms and relevance to the pathogenesis of polycystic ovary syndrome. J. Neuroendocrinol.

[11] Terán-Pérez G, Arana-Lechuga Y, Esqueda-León E, Santana-Miranda R, Rojas-Zamorano JÁ, Velázquez Moctezuma J (2012). Steroid hormones and sleep regulation. Mini Rev Med Chem.

[12] Bahammam A (2006). Does Ramadan fasting affect sleep?. Int J Clin Pract.

[13] Majlesi MB (1362). Bahar al anvar.

[14] Sheikh Sadooq Uyun Akhbar Al Reza.

[15] Roky R, Chapotot F, Hakkou F, Benchekroun MT, Buguet A (2001). Sleep during Ramadan intermittent fasting. J Sleep Res.

[16] Bogdan A, Bouchareb B, TouitouY (2001). TouitouY. Ramadan fasting alters endocrine and neuroendocrine circadian patterns. Meal-time as a synchronizer in humans?. Life Sci.

[17] Kumarapeli V, SeneviratneRde A, Wijeyaratne C (2011). Health-related quality of life and psychological distress in polycystic ovary syndrome: a hidden facet in South Asian women. BJOG.

[18] Sundararaman PG, Shweta, Sridhar GR (2008). Psychosocialaspects of women with polycystic ovary syndromefrom south India. J Assoc Physicians India.

[19] Zangeneh FZ, Naghizadeh MM, Abedinia N, Haghollahi F, Hezarehei D (2012). Psychological signs in patients with polycystic ovary syndrome. Journal of Family and Reproductive Health.

[20] Roelfsema F, Kok P, Pereira AM, Pijl H (2010). Cortisol production rate is similarly elevated in obese women with or without the polycystic ovary syndrome. J ClinEndocrinolMetab.

[21] Milutinović DV, Macut D, Božić I, Nestorov J, Damjanović S, Matić G (2011). Hypothalamic-pituitary-adrenocortical axis hypersensitivity and glucocorticoid receptor expression and function in women with polycystic ovary syndrome. Exp Clin Endocrinol Diabetes.

[22] Caraty A, Grino M, Locatelli A, Guillaume V, Boudouresque F, Conte-Devoix, Oliver C (1990). Insulin-induced hypoglycemia stimulate corticotropin-releasing factor and arginine vasopressin secretion into hypophyseal portal blood of conscious, unrestrained rams. Journal of Clinical Investigation.

[23] (2008). Acupuncture in Polycystic Ovary Syndrome: Current Experimental and Clinical Evidence. Journal of Neuroendocrinology.

[24] Sved AF, Cano G, Card JP (2001). Neuroanatomical specificity of the circuits controlling sympathetic outflow to different targets. Clin Exp Pharmacol Physiol.

[25] Zangeneh FZ, Mohammadi A, Ejtemaeimehr Sh, Naghizadeh MM, Aminee F The role of opioid system and its interaction with sympatheticnervous system in the processing of polycystic ovary syndrome modeling in rat. Arch GynecolObstet.

[26] Eyvazzadeh AD, Pennington KP, Pop-Busui R, Sowers M, Zubieta JK, Smith YR (2009). The role of the endogenous opioid system in polycystic ovary syndrome. Fertil Steril.

